# Human milk macro- and trace-elements: Simultaneous analysis in sub-milliliter amounts by ICP-MS and application to assessing acute supplementation effects

**DOI:** 10.1016/j.heliyon.2024.e34709

**Published:** 2024-07-16

**Authors:** Daniela Hampel, M. Munirul Islam, Setareh Shahab-Ferdows, Lindsay H. Allen

**Affiliations:** a Institute for Global Nutrition, Department of Nutrition, University of California, Davis, CA, 95616, USA; b USDA, ARS-Western Human Nutrition Research Center, Davis, CA, 95616, USA; c Nutrition Research Division, International Centre for Diarrhoeal Disease Research, Bangladesh (icddr,b), Mohakhali, Dhaka, 1212, Bangladesh

**Keywords:** Human milk, Macro-elements, Trace-elements, ICP-MS, Maternal supplementation, Bangladesh

## Abstract

Adequate concentrations of human milk (HM) nutrients, including macro- and trace-elements, are essential for healthy growth and development of exclusively breastfed (EBF) infants. To monitor potential risk of deficiencies, and evaluate the effects of interventions like supplementation, accurate analysis is crucial. Even recent methods reporting on HM macro- and/or trace-elements describe multiple methodological approaches and the need for several milliliters. We optimized and validated a comprehensive method for simultaneous analysis of 13 macro- and trace-elements for simultaneous analysis by inductively-coupled plasma-mass spectrometry. 100–600 μL HM were microwave digested with ≤1.5 mL HNO_3_ (70 %). The digest was diluted to 5 % final acid concentration. He-Kinetic Energy Discrimination (KED; Na, K, P, Ca, Mg, Fe, Cu, Zn, Cr, Mo) and O_2_-Dynamic Reaction Cell (DRC; As, Mn, Se) modes minimized remaining interferences. Accuracy (NIST SRM 1869 infant formula; n = 15, 4 weeks) varied from 93.2 to 103 % (CV: 2.8–8.5 %) with trueness ranging from 93.9 to 104 %. Inter-day variation of a HM-pool (n = 20, 3 weeks) varied between 4.1 and 8.5 % for most elements; Cr, Mo, Mn (all<5 μg L^−1^) had higher variation, up to 25 %. Analyzing HM from 18 Bangladeshi mothers (2–4 months postpartum; day 1 = baseline, n = 17; day 2/3 = supplementation, n = 21 each) revealed higher concentrations for P, Ca, and Zn post-supplementation (p < 0.05, Friedman's Chi-Square Test). Na, Mg, Zn, and Se had the highest number of samples (>80 %) with concentrations below the Adequate Intake. Our method allows for simultaneous and reproducible analysis of macro- and trace-elements with concentrations ranging over 6 orders of magnitude, without the need for separate analytics and sample preparations, and requiring only sub-milliliter amounts of HM. Additional elements may be included after optimization and validation. The results from Bangladeshi HM samples indicate selective supplementation effects and concerningly low concentrations for some elements, which could adversely affect the EBF infant.

## Highlights

• Exclusively breastfed infants rely on human milk for adequate minerals.
• We developed an ICP-MS method for 13 macro/trace-elements in human milk.
• Within one run we capture elements present in vastly different concentrations.
• We now can measure these key elements in minutes in low milk volumes.
• We found selected acute supplementation effects in milk from Bangladeshi mothers.

## Graphical abstract

**Figure:**
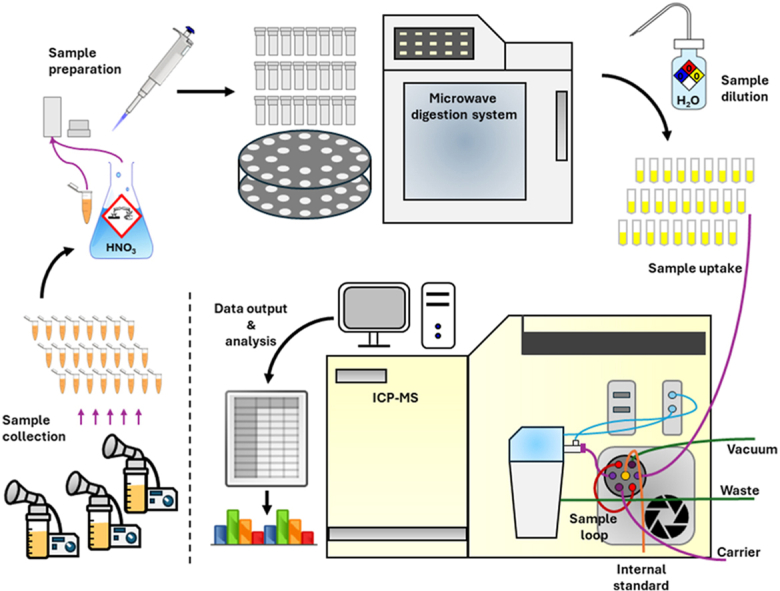

## 1 Introduction

Human milk (HM) is recommended as the single food source for infants up to 6 months of age [1]. Besides providing essential nutrients, HM also supports the gut microflora and various functions such as digestion and the endocrine and immune systems [2]. Hence, its composition and content are main driver of infant health, growth, and development. Macro- and trace-elements, co-factors for regulating enzymes, are essential for these physiological processes [[3], [4], [5]]. Some elements in milk are not affected by maternal dietary intake, while excess of essential trace elements, such as Fe, Cu, Zn, Se, or Mn can even reach toxic levels [6,7]. Hence, to better understand the link between HM macro- and trace-elements and infant requirements for healthy outcomes, their accurate analysis in human milk is indispensable [8].

The analysis of HM-elements has been well-described using methods such as atomic absorption spectroscopy (AAS) [[9], [10], [11], [12]] or inductively coupled plasma–atomic emission spectroscopy (ICP-AES) [2,[13], [14], [15], [16], [17]]. ICP-MS (mass spectrometry), however, has emerged as the method of choice [2,3,5,6,13,[18], [19], [20], [21], [22], [23], [24]]. Other methods more recently described include X-ray fluorescence or total reflection X-rays fluorescence (TRXF) spectrometry [25,26].

Biological materials, such as human body fluids, can be problematic for ICP-MS analyses due to the presence of considerable amounts of proteins, inorganic salts, and small organic molecules [27]. HM further contains considerable amounts of sugar and lipids, which adds a layer of complexity by producing higher amounts of polyatomic interferences during digestion. These interferences can impact the validation process, e.g. by interfering with the analyte recovery determination. Further, a certified HM standard material is not available to confirm accuracy for validation.

Several sample preparations for ICP-MS analysis have been described including the more recently introduced use of alkaline solutions consisting of numerous reagents [2,3,20,22], some of which are acutely toxic or hazardous to health. Alternatively, acid digestion has been traditionally used by subjecting the sample to concentrated nitric acid (HNO_3_) often in the presence of hydrogen peroxide (H_2_O_2_) followed by microwave [18,19], water bath, or hot plate digestion [21,23]. Several reports describe the need for sample volumes of ≥1 mL [18,24,28,29], and given their vastly different concentrations in HM, macro- and trace elements have been commonly measured by two separate approaches [2,3,13].

While ICP-MS provides superior sensitivity, multi-element capabilities, a wide linear dynamic range, and isotope measurements [30], it is also susceptible to spectral, polyatomic interferences, which can significantly impact the results. These interferences are often ions generated from the plasma and/or sample and possess identical mass-to-charge ratios to the analyte ions. The introduction of the universal cell enables the use of inert (He) or reactive gases (NH_3_, O_2_, H_2_) in kinetic energy discrimination (KED) and dynamic reaction cell (DRC) modes, respectively, and has been successfully employed to overcome these challenges [31].

Here, we report an ICP-MS method that enables the rapid analyses of selected HM macro- and trace-elements simultaneously, eliminating the need for multiple techniques for the same data, and requiring only limited sample volume and a minimal set of reagents. We further compared results obtained with the newly optimized method to measurements obtained by TRXF. The developed method was used for elemental analysis of milk from Bangladeshi mothers, 2–4 months of lactation, who were part of the Breast-Milk-Quality (BMQ) study [32], to examine acute effects of maternal supplementation.

## 2 Materials and methods

### 2.1 Chemicals, reagents, and other materials

NexION Setup Solution (1 μg L^−1^) for daily smart tune procedures and TruQ^TM^ms Internal Standard Mix (Bi, Ge, In, Li^6^, Sc, Tb, Y in 5 % nitric acid) were purchased from PerkinElmer (Waltham, MA, USA). Concentrated nitric acid (HNO_3_, ∼70 %), trace-element grade, was obtained from Fisher Scientific (Waltham, MA, USA). Analytical standards for single elements were obtained from AccuStandard (New Haven, CT, USA; Na, K, Mg, Ca: 10,000 μg/mL in 2–5% HNO_3_; P: 10,000 μg/mL in water; Fe, Cu, Zn, Cr, Se: 1000 μg/mL in 2–5% HNO_3_; Mo, Mn, As: 100 μg/mL in 2–5% HNO_3_). Triple de-ionized water was available in-house using a Barnstead E-PURE 3-Module water purification system (APS Water Services Corporation, Lake Balboa, CA, USA). 15 and 50 mL Corning™ polypropylene centrifuge tubes, used for sample dry bath heat treatment and external standard curve preparation, respectively, and 15 mL Sarstedt conical bottom skirted polypropylene tubes used as secondary container for standards, were obtained from Fisher Scientific.

### 2.2 Equipment

#### 2.2.1 ICP-MS

A NexION 2000P ICP-MS system (PerkinElmer; Waltham, MA, USA) coupled with a DC-4DXS autosampler (Elemental Scientific; Omaha, NE, USA) and a WhisperCool 1 HP chiller (PolyScience; Niles, IL, USA) was used for all experiments. A 2.5 mL sample loop for FAST valve injection ensured sufficient sample volume for analysis of all elements. The All Matrix Solution (AMS) system was employed to reduce matrix suppression [33]. Poly-atomic interferences were minimized using Universal Cell Technology™, employing kinetic energy discrimination (KED, helium) and dynamic cell reaction (DRC, oxygen) modes. Daily smart tune was performed to ensure all parameters were met for accurate analysis. The simultaneous analysis of macro- and trace-elements was possible using dual detector mode, which was tuned every day after successful smart tune. Detector voltages were tuned monthly.

#### 2.2.2 Sample digestion system

Sample digestion was carried out using a MARS6 microwave digestion system (CEM Corporation; Matthews, NC, USA) equipped with a CEM MARSXpress™ vessel assembly (10 or 20 mL). The assembly allowed for up to 40 digestions simultaneously. Sample digestion was initially tested using a Fisherbrand™ Isotemp™ digital dry bath accommodating 4 x 12 block heaters (Fisher Scientific; Waltham, MA, USA).

#### 2.2.3 Human milk samples and controls (QCs)

Pooled human milk was kindly provided by a single donor in the Sacramento, CA, area, and used for initial sample preparation testing and then for quality control (QC) and monitoring during routine analysis. Additionally, NIST Standard Reference Material (SRM) 1869 infant formula (IF; National Institute of Standards and Technology; Gaithersburg, MD, USA) with certified concentrations of the macro- and trace-elements was used for method development, validation, and QC during routine analysis.

Available HM samples (full breast expression, n = 59), collected for the Breast Milk Quality (BMQ) study, from 18 apparently healthy Bangladeshi mothers at 2–4 months postpartum, collected midday to early afternoon (12:00PM to 3:00PM) on 3 consecutive days (day 1: baseline, n = 17; day 2: 1 x multiple micronutrient supplement (MMS, n = 21); day 3: 2 x MMS, n = 21), were analyzed with the developed method to examine a) potential acute supplementation effects, and b) Na:K ratios as indicator of subclinical mastitis. Samples were collected between October 2013 and February 2014 (NTC02756026). Study details, including the supplementation regimen, have been previously reported [32].

#### 2.2.4 Analytical procedures

##### 2.2.4.1 ICP-MS

Up to 600 μL HM were transferred to a 20 mL digestion vessel and mixed with 1.5 mL HNO_3_ (70 %) for a final volume of 2.1 mL. Volumes were chosen to fulfill the minimum volume requirements for digestion of 2.0 mL, and to enable acid dilution to acceptable concentrations for analysis. Lower sample volumes were suitable for analysis but required additional water to fulfill the minimum aqueous volume of 600 μL.

After about 15 min incubation at room temperature the vessels were closed, transferred into the carousel, and digested. Within 20–25min, the samples were heated to 205 °C and held for 15 min before cooling to below 125 °C. After the samples were further cooled closer to room temperature to reduce the pressure inside the vessel, the samples were opened in a chemical fume hood, diluted to 20 mL using triple-deionized water, and 10–14 mL were transferred to a 15 mL centrifuge tube for analysis. Each sample digestion batch consisted of 1 blank, 2 controls (NIST SRM 1869 infant formula, pooled human milk), and 37 milk samples for a total of 40 vessels. Vessels were cleaned after each digestion by adding 4 mL concentrated HNO_3_ (70 %) following the procedures of the “Xpress clean” program according to the manufacturer.

##### 2.2.4.2 S4 T-Star high performance TXRF

The QC-HM as well as another HM-pool consisting of milk from several apparently healthy women in the Vancouver, BC area (UBC-pool), and a single donor in the Sacramento, CA area (LP) were used to compare results obtained with our method to results from the same samples obtained using the S4 T-Star high performance TXRF (total reflection X-rays fluorescence) spectrometer for ultra-trace element analysis (Bruker, Billerica, MA, USA). The analysis was carried out at Bruker Madison, WI, USA. The system can be used in a wide range of sample types, including biological samples such as body fluids, and is equipped with an automatic quality control feature for quality control and assurance [34]. 1 mL of human milk sample were placed into a reaction vial and diluted with 1.5 mL ultrapure water. Then, 10 μL V (1g L^−1^), 10 μL Ga (100 mg L^−1^), and 5 μL Pd (1g L^−1^) were added as internal standards. After homogenization, 10 μL of the sample was transferred ono a quartz glass carrier and analyzed after a drying step under vacuum. Quantitative measurements were taken using Mo–K, W-L, and W-Brems excitation (all 1000s).

#### 2.2.5 Data analysis

##### 2.2.5.1 Method validation

Since no SRM is available for human milk, NIST 1869 SRM was used for method optimization. Recovery rates (R%) and Trueness (T%) were determined by 15 measurements on 15 days, similar to Refs. [21,35].

(I) R(%) = C_certified_*100/C_found_

Where R(%) is the recovery rate in percent, C_certified_ is the certified value by NIST, and C_found_ is the measured value (macro-elements Na, K, P, Ca, and Mg in mg L^−1^, remaining trace-elements in μg L^−1^).

Trueness was obtained using z-scores to calculate the difference between measured and certified values as previously described [35].

(II) *z* = (*X*_found_ – *X*_certified_)/sqrt[(SD_found_/n_found_)+(U_95_/2)^2^]

where *X*_found_ is the mean concentrations (mg L^−1^ or μf L^−1^) and SD_found_ is the standard deviation of measured analytes in the NIST 1869 SRM, *X*_certified_ is the mean analyte concentrations as certified by NIST, U_95_ refers to the 95 % confidence limits of the concentrations of the SRM, and n_found_ is the number of measurements (15 replicates). A z-score ≤2 signifies the measured value is true to the certified concentrations. A negative z-score indicates that the measured concentration was lower than the certified value, resulting in values < than 100 %.

The limit of detection (LOD) was calculated as three times the standard deviation (SD) of 10 replicates of a blank sample, which comprised of reagents (HNO_3_ and water) but no matrix. The limit of quantitation (LOQ) was estimated by using 10 times SD of the same blank replicates.

The goodness of fit of the standard curves was evaluated by simple linear regressions using 10 standard curves analyzed on 10 different days within 2 weeks. Further, nominal concentrations were back-calculated and simple linear regression as employed to asses slopes, coefficients of correlations (r^2^), deviation from linearity, and whether slopes and intercepts differ. All calculations were carried out using Graphpad Prism, version 10.2.3 (GraphPad Software, Boston, MA, USA) or Excel for Microsoft 365 (Microsoft Corporation, Redmond, WA, USA).

Lastly, for elements that were not detectable in the HM-QC, or only present in negligible amounts below the limit of quantitation (LOQ), samples were spiked with Cr, Mn, Mo, and As. This standard experiment at two levels in the low μg L^−1^-range (1.88/3.75–3.75/7.5 μg L^−1^) was carried out to test the goodness of the optimized conditions for these elements in HM.

##### 2.2.5.2 Statistical analysis of Bangladeshi sample set

Descriptive statistics for the validation (mean, standard deviation (SD), coefficient of variance (CV)) and Na:K-ratios, median, and range were calculated using Excel for Microsoft 365.

The Wilcoxon matched-pairs signed rank test was used to compare results obtained with IPC-MS and S4-T-Star (GraphPad Prism). All other statistical analysis was carried out using SAS for Windows 9.4 (SAS, Cary, NC, USA). Treatment effects in HM from Bangladeshi mothers were tested using Friedman's Chi Square test for non-parametric data using the “proc freq” procedure. A pairwise comparison (day 1 vs 2, 1 vs 3, 2 vs 3) was carried out when treatment effects were significant (*p*-values <0.05), using the same “proc freq” statement including a “where” statement to specify the pair. Proc means procedure was used to calculate medians, interquartile range (IQR), and frequency of samples with concentrations below those used to set the Adequate Intakes (AIs) for infants up to 6 months [[36], [37], [38], [39]].

## 3 Results

### 3.1 Sample preparation and analysis

**Fig. 1:**
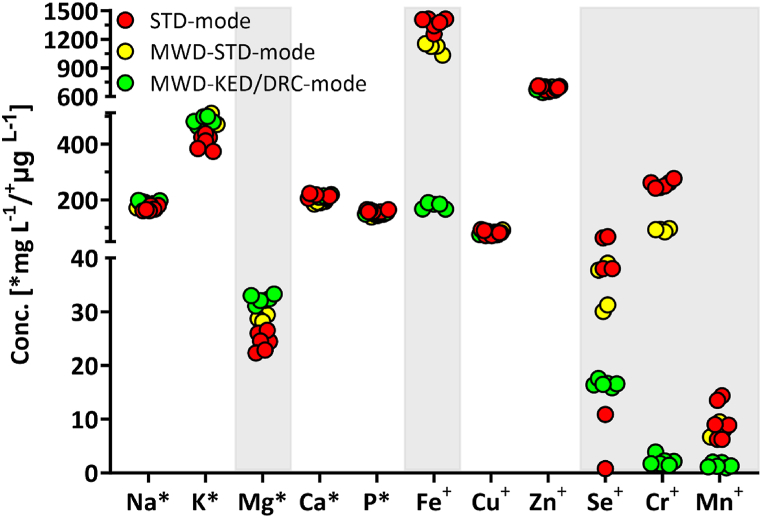
Effects of polyatomic interferences and ion suppression on concentrations of macro- and trace elements in human milk. STD: standard. MWD: microwave digestion. KED: kinetic energy discrimination. DRC: dynamic reaction cell.

Acid digestion of a HM-pool showed initially promising results and was therefore used, while in our hands the alkaline solution treatment [22] produced an opaque sample not appropriate for analysis (data not shown). A 2h at 90 °C heat treatment using a dry bath was found to be suitable for digestion tests as longer heat exposures provided comparable results (data not shown). H_2_O_2_ as an additive during acid digestion did not yield superior results (data not shown) and was therefore omitted. Employing microwave digestion was superior compared to dry bath heat incubation removing more interferences for Se, Cr, and Fe. However, best results were obtained after microwave digestion and the optimized ICP-MS parameters, employing AMS and collision (KED) and reaction cell (DRC) modes (Fig. 1). Given the unavailability of HM with certified concentrations for the analytes, ICP-MS parameters were optimized using NIST SRM 1869 IF. Optimized parameters are presented in Table 1.

**Table 1:** ICP-MS operating conditions (PerkinElmer NexION 2000).

Flow Parameters (L min^−1^)		Other	
Plasma gas flow	15	RF power (W)	1600
Auxiliary gas flow	1.2	Analog stage voltage (W)	1600
Nebulizer gas flow	0.94	Pulse stage voltage (W)	−2100
AMS gas flow	0.15	Sampling dept (mm)	147
**He-KED**		**O**_**2**_**-DRC**	
Helium flow (mL min^−1^)	5.3	Oxygen flow (mL min^−1^)	1.5
RPq	0.25	RPq	0.25–0.65
Internal standard	^115^In,^159^Tb	Internal standard	^61^ScO,^90^GeO
Analytes - ME	^23^Na,^24^Mg,^31^P,^39^K,^43^Ca,	Analytes - TE	^71^MnO,^91^AsO,^94^SeO
Analytes - TE	^52^Cr,^56^Fe,^63^Cu,^66^Zn,^98^Mo		
**Processing**		**Sampling**	
Replicates	3	Sample flush (rpm)	−35
Reading/replicate	1	Read delay (s)	18
Sweeps/reading	50	Analysis (rpm)	−22
Detector	dual	Wash (rpm)	−35
Blank subtraction	After IS		
QID	on		

### 3.2 Method validation

#### 3.2.1 Inter-day variation, precision, trueness

The trueness of the 15 measurements (4 weeks, n = 15) for NIST SRM 1869 IF (SRM) revealed a range of 93.9–104 % from the certified SRM value. Inter-day variation expressed as coefficient of variation (CV) for the SRM during the validation process and a HM-pool during routine analysis (HM-QC; 3 weeks, n = 20) was ≤8.5 % for most elements (Table 2). Ultra-trace elements (usually <5 μg L^−1^; HM-Cr, Mo, Mn) revealed higher variations when measured in HM (<25 %), indicating these elements were present only in amounts below the limit of quantitation. In fact, monitoring these elements during routine analyses revealed that the CV of the triplicate measurements during an analytical run was consistently below 10 % when present at a specific threshold (Cr: 3 μg L^−1^, Mo: 0.5 μg L^−1^, As: 0.5 μg L^−1^, Mn: 1.5 μg L^−1^). Arsenic, not present in the NIST SRM 1869 IF or the HM-QC, was nonetheless found in some samples during routine analysis with reproducible sub- μg L^−1^ measurements. Standard addition experiments for these elements in HM showed recoveries for all between 93.7 and 104.6 % with CV below 5 % (Table 3).

**Table 2:** Accuracy and precision of NIST SRM 1869 infant formula for the target elements and goodness of standard curves^a^.

Element	NIST SRM 1869				Human milk	LOD^f^	LOQ^g^	Slope^h^ (±SD)	(r^b^)^i^ (CV)
	C_Theory_ (U_95_)^b^	C_analyzed_ (±SD)^c^	A & P^d^	T(%)^e^	C_analyzed_ (CV)^e^				
n		15			20	10			
Na	86.9 (2.5)	83.8 ±3.5	96.5 (4.1)	97.7	170 (4.2)	0.026	0.086	1.000 ±<0.001	0.998 (0.10)
Mg	43.9 (0.46)	42.0 ±2.0	95.8 (4.8)	95.8	29.6 (4.1)	0.005	0.016	0.997 ±0.006	1.000 (0.01)
P	193.7 (2.6)	188.9 ±15.3	97.5 (8.1)	97.1	146 (7.4)	0.028	0.093	0.999 ±0.001	1.000 (0.02)
K	349.8 (5.1)	360.3 ±30.6	103 (8.5)	104	459 (6.5)	0.047	0.155	0.999 ±0.001	1.000 (0.07)
Ca	211.0 (6.0)	213.7 ±15.8	101 (7.4)	101	196 (5.8)	0.028	0.093	1.000 ±<0.001	1.000 (0.01)
Fe	7620 (171)	7375 ±355	96.8 (4.8)	97.1	157 (8.5)	0.712	0.780	1.000 ±<0.001	0.999 (0.06)
Cu	879 (17.6)	844 ±23.7	96.0 (2.8)	96.0	72.3 (7.5)	0.023	0.048	1.000 ±<0.001	1.000 (0.22)
Zn	6663 (148)	6209 ±285	93.2 (4.6)	93.9	635 (5.9)	0.724	1.22	1.000 ±<0.001	1.000 (0.01)
Se	37.3 (3.8)	40.2 ±2.0	108 (4.9)	102	18.1 (5.6)	0.001	0.003	1.000 ±<0.001	0.999 (0.05)
Cr	39.7 (3.1)	41.5 ±3.4	104 (8.2)	101	2.4 (23.9)	0.021	0.031	1.000 ±<0.001	1.000 (0.01)
Mn	2128 (74)	2025 ±74	95.2 (3.7)	97.2	1.5 (14.6)	0.025	0.024	1.000 ±<0.001	0.998 (0.04)
Mo	74.6 (2.2)	71.3 ±2.9	95.6 (4.0)	97.2	0.48 (18.4)	0.003	0.005	1.000 ±<0.001	1.000 (0.01)
As	n/a	n/a	n/a	n/a	n/a	0.011	0.010	1.000 ±<0.001	0.998 (0.05)

a Macro-element concentration and LOD/LOQ (Na, Mg, P, K, Ca) in mg L^−1^, trace-elements (Fe, Cu, Zn, Se, Cr, Mn, Mo, As) in μg L^−1^.

b C_Theory_: Certified concentration by NIST; U_95_: expanded uncertainty of the certified value.

c C_analyzed_: Measured concentration ± SD.

d A & P: Inter-day accuracy and precision in % (CV).

e Trueness (T%) calculated based on Taverniers et al. [35] using the 15 inter-day measurements. Since the mean and SD from all measurements is used, only one value for trueness is available.

f LOD: limit of detection, 3xSD of blank (n = 10) concentrations.

g LOQ: limit of quantitation, 10xSD of blank (n = 10) concentrations.

h Slope (mean ± SD) of 10 measurements (back-calculation to the nominal standard concentrations).

i Mean coefficient of determination (r^2^) (CV) of 10 analytical standard curve runs.

**Table 3:** Standard addition for Cr, Mo, Mn, and As in human milk.

Element	Level 1 (μg L^−1^)	Accuracy (CV), %	Level 2 (μg L^−1^)	Accuracy (CV), %	Overall accuracy (CV), %
Cr	3.75	101 (0.3)	7.50	105 (4.2)	103 (2.5)
Mo	1.88	93.7 (0.8)	3.75	94.4 (0.4)	94.0 (0.5)
As	1.88	97.8 (0.2)	3.75	97.6 (1.3)	97.7 (0.1)
Mn	1.88	101 (0.7)	3.75	97.2 (0.6)	98.9 (2.4)

^1^ CV: Coefficient of variation.

#### 3.2.2 Detection limits and linearity

Detection limits (LOD and LOQ, Table 2) for all analytes were well below the typical range of their concentrations in human milk. Analysis of the 10 standard curves for each analyte showed that there was no deviation from linearity, with mean coefficients of determination (r^2^) greater than 0.997 for all analytes (Table 2). All mean slopes of the back-calculated nominal concentrations ranged between 0.997 and 1.000, which no significant differences in the slopes or intercept (all p < 0.05). All curves (standard curves and curves of the back-calculated nominal concentrations) did not significantly deviate from linearity (all p < 0.05).

### 3.3 ICP-MS vs. S4 T-star TXRF spectrometer comparison

**Fig. 2:**
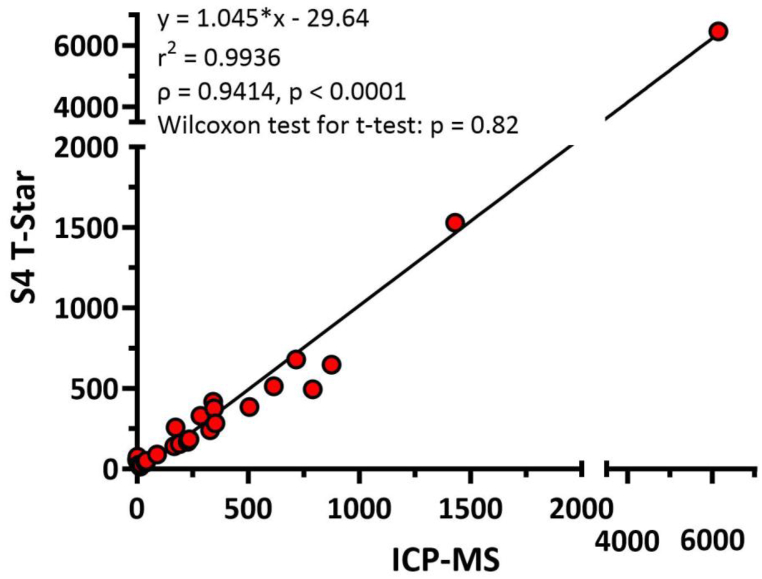
x-y-plot of concentrations^1^ of human milk macro- and trace elements analyzed by ICP-MS against the same measurements by S4 T-Star TXRF spectrometer^2^. ^1^ Macro-element concentration (Na, Mg, P, K, Ca) in mg L^−1^, trace-elements (Fe, Cu, Zn, Se, Cr, Mn, Mo, As) in μg L^−1^. ^2^ Data included are concentration of Mg, P, K, Ca, Cr, Fe, Cu, Zn, Se, Mn. As was not detected in the samples by either method, and Na and Mo were not detected using TXRF.

ICP-MS analyses were conducted in duplicate, while the S4 T-star measurements were done in triplicate. Average concentrations were used for comparison. Elements were excluded if they were not detected in one of the analytical approaches (ICP-MS: As; S4 T-Star: Na, Mo, As), which resulted in 28 data points. The XY-plot (ICP-MS vs. S4 T-Star) revealed good linearity with a slope of 1.045 and a goodness of fit (r^2^) of 0.9936 (Fig. 2). The Wilcoxon matched-pairs signed rank test indicated no significant difference between the measurements from the two methods (p > 0.82) with a significant correlation (δ) of 0.94 (p < 0.0001).

### 3.4 Human milk analysis

Analyzing HM samples from Bangladeshi mothers showed that P, Ca, and Zn concentrations on day 2 were significantly higher than on day 1 but comparable to day 3. Mg concentrations on day 2 were comparable to day 1 and day 3, but at day 3, concentrations were significantly higher than at day 1 (all p < 0.04). All other measured macro- and trace-elements had comparable concentrations on all 3 days (Table 4). When comparing our results to the Adequate Intake (AI) for infants up to 6 months, most of the Na and Se concentrations were below the AI recommendations (>85 %; Table 5). For many elements, however, the AI was met by more than 75 % on all days, and for some all samples reached or exceeded their AI value (P, Cr, Mo). The significant changes in concentrations were also reflected in the AI comparison, e.g., Ca concentrations were below AI in about 6 % of the samples on day 1, but all samples met the AI on day 2 and 3. The higher Zn concentrations with maternal supplementation reduced the number of samples not meeting AI values from 82.5 % to 62 % over the 3-day period. Only Mg showed comparable sample numbers below AI on days 1 and 3, even though the milk concentrations were significantly higher on day 3 vs. day 1(p = 0.036). Na/K ratios, an indicator for subclinical mastitis, ranged from 0.21 to 0.54 (Table 4) and therefore fell below the cut-off of 0.6 suggested as the cut-off for mastitis [40].

**Table 4:** Median concentrations and interquartile ranges (mg L^−1^, μg L^−1^)^a^ of macro- and trace-elements, and the Na:K-ratio (median and range) in milk from Bangladeshi mothers at 2–4 months postpartum.^b^.

Element	Day 1	Day 2	Day 3	p-value^c^
Na	104 (94.8, 119)	110 (100, 129)	119 (110, 127)	0.1
Mg	34.1 (33.0, 35.5)^a^	37.1 (33.3, 43.5)^a,b^	36.1 (32.6, 39.6)^b^	0.06
P	194 (166, 208)^a^	208 (189, 230)^b^	206 (195, 227)^b^	**0.031**
K	590 (519, 630)	601 (533, 663)	569 (536, 633)	0.6
Ca	313 (283, 348)^a^	336 (313, 354)^b^	340 (330, 370)^b^	**0.014**
Fe	414 (358, 506)	423 (326, 570)	409 (328, 569)	0.5
Cu	374 (331, 523)	420 (345, 513)	430 (382, 458)	0.5
Zn	1807 (1167, 2169)^a^	1900 (1384, 2776)^b^	2144 (1216, 2858)^b^	**<0.001**
Se	10.3 (8.6, 12.2)	10.7 (9.9, 12.5)	11.1 (9.6, 13.1)	0.6
Cr	15.7 (10.5, 21.6)	14.5 (10.7, 19.7)	18.2 (12.4, 25.3)	0.5
Mn	7.78 (6.4, 8.8)	8.24 (6.2, 11.8)	11.3 (6.9, 27.6)	0.1
Mo	3.30 (1.6, 5.8)	2.55 (1.8, 7.6)	3.25 (1.8, 7.3)	0.7
Na:K ratio^d^	0.34 (0.21–0.54)	0.33 (0.22–0.47)	0.36 (0.25–0.44)	0.06

a Macro-element concentration (Na, Mg, P, K, Ca) in mg L^−1^, trace-elements (Fe, Cu, Zn, Se, Cr, Mn, Mo, As) in μg L^−1^.

b Day 1: no supplement, day 2: 1x multiple micronutrients (MMN), day 3: 2x MMN. Significant differences (p < 0.05; Friedman's Chi Square test and pairwise comparisons) are indicated by different superscript letter. No superscript indicates no significant differences.

c p-values obtained from Friedman's Chi Square test to evaluate treatment effects.

d Na:K-ratio: molar ratio of sodium to potassium concentrations to assess clinical mastitis.

**Table 5:** Number (n) and percent of milk samples from Bangladeshi mothers that did not meet the Adequate Intake values.

Element	AI^a^	Day 1	Day 2	Day 3
		Sample < AI, n (%)		
Na	140	15 (88.2) 104 (67.1–134)^b^	20 (95.2) 110 (76.0–136)	20 (95.2) 118 (91.2–139)
Mg	40	14 (82.4) 33.9 (21.3–38.2)	14 (66.7) 34.1 (22.6–39.9)	17 (81.0) 33.8 (25.8–39.8)
P	130	0 (0.0) –	0 (0.0) –	0 (0.0) –
K	515	3 (17.6) 490 (453–513)	4 (19.0) 496 (478–507)	4 (19.0) 494 (485–510)
Ca	255	1 (5.9) 219	0 (0.0) –	0 (0.0) –
Fe	345	4 (23.5) 302 (197–314)	6 (28.6) 307 (254–326)	6 (28.6) 286 (274–328)
Cu	256	1 (5.9) 218	2 (9.5) 224,237	1 (4.8) 185
Zn	2564	14 (82.4) 1372 (839–2252)	15 (71.4) 1577 (995–2559)	13 (81.0) 1527 (953–2310)
Se	19	17 (100) 10.3 (6.6–15.2)	21 (100) 10.7 (7.6–16.4)	21 (100) 11.1 (8.4–15.7)
Cr	0.256	0 (0.0) –	0 (0.0) –	0 (0.0) –
Mn	3.846	1 (5.9) 3.4	0 (0.0) –	1 (4.8) 3.6
Mo	0.256	0 (0.0) –	0 (0.0) –	0 (0.0) –
As	–			

a AI: Adequate intake recommendations for infants 0–6 months [[36], [37], [38], [39]]. Macro-element (Na, Mg, P, K, Ca) concentrations in mg.^L−1^, trace-elements (Fe, Cu, Zn, Se, Cr, Mn, Mo, As) in μg L^−1^. Day 1: no supplement, day 2: 1x multiple micronutrients (MMN), day 3: 2x MMN.

b Median concentrations (range) of values below AI for each element and day. Days on which only 1 or 2 value < AI, values are displayed.

## 4 Discussion

### 4.1 Sample preparation and analysis

While ICP-MS is a powerful tool for elemental analysis, removal or minimization of spectral interferences remains challenging. Typical interferences include polyatomic species, double charge ions, or isobaric interferences, which are prominent in complex fluids such as HM [21]. Even the type of sample preparation can affect the level of interference. Microwave digestion using sealed vessels has been an excellent choice for trace-element analysis, enabling speedy and complete digestion. Microwave systems can heat samples instantly exposing the sample to extreme pressures, which speeds up the sample decomposition to complete digestion, overcoming the known drawbacks of heating block digestions such as prolonged heating times and inferior digestion quality [41]. Our comparison of microwave and heating block digestions aligns with these findings. Spectral interferences were already reduced by microwave digestion for Fe, Cr, and Se, most likely due to the complete sample matrix degradation. This efficient sample digestion technique in conjunction with the capabilities of the ICP-MS, using AMS and the universal cell, allowed for reproducible and accurate analysis of the selected target analytes.

### 4.2 Method validation

Since human milk with certified values for macro- and trace-elements was unavailable, we used the SRM for our optimization process. Under our optimized conditions, all target analytes were recovered over 93 % of the theoretical value with a trueness of at least 93.9 %, with trueness ranging from 93.9 to 104 %, indicating accurate and reproducible analysis. Using a HM-pool, the coefficient of variance (CV) was still below 10 % for most of the target analytes; higher CVs were observed only for ultra-trace elements (Cr, Mo, Mn). The higher CVs are likely a function of very low concentrations present in the milk, which required measurements in the ppt-range. As already mentioned, arsenic (As) was not present in the IF or the HM-pool. Nevertheless, the standard experiments carried out for these ultra-trace elements and As confirmed that they are indeed reliably analyzable in HM under the optimized conditions when present in low concentrations.

While standard addition experiments were used for these particularly low-abundant or absent elements, they were not considered for elements that are present in the matrix at reproducibly measurable concentrations. ICP-MS interferences are driven by the mass to charge ratio chosen for analysis, the degree of digestion completion, and the choice of detection mode (e.g., STD, KED, DRC). Hence, the interferences can be considered constant under the chosen conditions, which won't affect the recovery of the spiked concentrations. Hence, when estimating the recovery by comparing theoretical values to measured values obtained from spiked and non-spiked samples, the level of interferences does not affect the relative recovery. As a result, the elements are recovered close to 100 % without any insight into true accuracy, as the effects of the interferences are not captured.

### 4.3 ICP-MS vs. S4 T-star TXRF spectrometer comparison

Both approaches for elemental analysis provided comparable results in 3 different HM samples, indicated by a trendline slope of 1.05, a non-significant Wilcoxon matched paired signed rank test and a significant pairing efficiency. TXRF is a special energy-dispersive x-ray analytical technique [42], while ICP-MS uses argon plasma to ionize the sample into positively single-charged ions, which are then extracted into the mass analyzer [43]. Our results show that, at least for the included elements, both techniques -while fundamentally different-yield comparable results, emphasizing further that our optimization using the NIST IF is indeed applicable to the HM matrix producing accurate and reliable results.

TXRF offers low limits of detection and discriminates against matrix, which results in very low background noise. It is easy to perform and requires low sample volumes. ICP-MS is highly sensitive and specific, offering very low detection limits and isotopic information [43,44]. Both techniques also have drawbacks, such as the previously mentioned spectral interferences in ICP-MS, or a potentially less robust calibration method in TXRF [43,45]. Here, TXRF did not provide any results for sodium or iodine in any of the milk samples analyzed, although both elements are typically present in HM. The presence of sodium was further confirmed by our ICP-MS analyses. Indeed, due to absorption errors, light elements such as Na or lighter, are not accurately analyzable [45,46]. Hence, it is important to understand the analytical needs to identify a suitable technique. Since our interest included results for Na, TXRF was not suitable for our purpose.

### 4.4 Human milk analysis

Maternal supplementation and/or intake is thought to have no effect on most of the analyzed elements; only Se has been shown to respond to supplementation, while no data was found for P and Mo [[47], [48], [49], [50], [51]]. Here, we observed acute supplementation effects for milk P, Ca, Mg, and Zn. Although our results agree with the literature for many of the analyzed elements it is noteworthy that we only examined acute supplementation effects, while the literature reports on effects of long-term supplementation and maternal (dietary) intake, therefore our results may not be directly comparable but rather complementary by providing further insight into the transfer of elements into milk.

Comparing our results obtained at day 1 (median concentrations, no supplement) with values from studies conducted in Europe, Australia, Indonesia, and Japan, showed comparable milk concentrations for Na, Mg, and Se [2,3,24,52,53] while P, K, Ca, Cu, Zn, and Mo concentrations tended to be higher in our sample set. Cr (22-fold), Mo (7-fold), and Fe (4-fold) displayed a wide range of values across the different studies. Cu and Zn data from another Bangladeshi study revealed lower concentrations for both trace elements compared to our results [54]. Several factors may have impacted these variations, such as demographics and environment, different analytical approaches, or different lactation stages during sample collection.

When comparing our results to the current AI recommendations [36,37,39] we found that the prevalence of values below the AI was over 80 % for Na and Se throughout the study, while supplementation reduced the prevalence of low values for Zn from 82.4 to 61.9 % over the 3-day study. Mg analyses revealed a similar high prevalence of inadequate values as Na and Se on days 1 and 3, while on day 2 the prevalence was considerably lower. These findings do not reflect the results of the pairwise comparison for Mg, showing a trending significant difference between days 1 and 2 and significantly higher concentrations for day 3 v. day 1. This may be due to the fact that the AI-comparisons are carried out using a cut-off while the pairwise comparison uses continuous variables, which will capture the overall trend in concentrations, even if higher concentrations with supplementation fall below the AI cut off. The remaining elements showed prevalences ranging from 0 to 30 % for all study days, indicating adequate supply for the EBF infant for some of the analyzed elements.

Lastly, the Na:K-ratio in all of our samples was below 0.6, a commonly used cut-off for indicating subclinical mastitis, an inflammation of breast tissue that most often occurs in the first 2–3 weeks of lactation but appears at about 3 months in 10 % of lactating women [40,55]. Hence, none of the participants appeared to experience breast inflammation due to mastitis, which may result in decreased milk production and is linked with poorer rates of non-EBF and early weaning [56].

## 5 Conclusion

Our newly developed ICP-MS method for selected HM macro- and trace-elements enables the simultaneous analysis of the described elements in sub-milliliter amounts in spite of concentration differences ranging over 6 orders of magnitude. Microwave digestion and KED/DRC modes are necessary to accurately measure the target analytes. The accuracy of our method is further supported by the high level of agreement in concentrations measured by our method and TRXF. Our method is expandable to include additional elements after an optimization and validation process. Using our approach, we could identify low concentrations for some elements as well as selected acute supplementation effects in milk from Bangladeshi mothers, data that provides further insight into the effects of maternal supplementation on milk macro- and trace-elements.

## Ethical approval

The BMQ Study was approved by the Institutional Review Board of the University of California, Davis (IRB ID 429296-1, approved November 03, 2013), and the icddr,b internal review board. The trial was registered at clinicaltrials.gov NCT02756026.

## Data availability

Data will be made available upon request.

## Funding

This work was supported by the 10.13039/100000865Bill & Melinda Gates Foundation, Seattle, WA [OPP1148405/INV-002300, OPP1061055]; and 10.13039/100000199USDA intramural funds [2023-51530-025-00D].

## CRediT authorship contribution statement

**Daniela Hampel:** Conceptualization, Formal analysis, Investigation, Methodology, Validation, Writing – original draft, Writing – review & editing. **M. Munirul Islam:** Investigation, Project administration, Supervision, Writing – review & editing. **Setareh Shahab-Ferdows:** Conceptualization, Investigation, Writing – review & editing. **Lindsay H. Allen:** Conceptualization, Funding acquisition, Writing – review & editing.

## Declaration of competing interest

The authors declare that they have no known competing financial interests or personal relationships that could have appeared to influence the work reported in this article.
